# Impact of chemotherapy on breast cancer−related lymphedema: a retrospective study

**DOI:** 10.3389/fonc.2026.1787021

**Published:** 2026-04-27

**Authors:** Liqin Wang, Yuemin Weng, Weiqi Wei, Jun Bu, Cici Zhang

**Affiliations:** 1 Health Management Center (Preventive Treatment) of Baiyun Hospital, Guangdong Provincial Second Hospital of Traditional Chinese Medicine (Guangdong Provincial Engineering Technology Research Institute of Traditional Chinese Medicine), Guangdong Provincial Key Laboratory of Research and Development in Traditional Chinese Medicine, Guangzhou, China; 2 Department of Radiology, Guangzhou Red Cross Hospital, Guangzhou, China

**Keywords:** breast cancer, chemotherapy, factors, lymphedema, neoadjuvant chemotherapy

## Abstract

**Objective:**

This study aimed to investigate the impact of chemotherapy on the risk of breast cancer-related lymphedema (BCRL), with a specific focus on effects of different regimens, treatment duration (number of cycles), and timing of chemotherapy.

**Methods:**

In this retrospective study, 201 patients with pathologically confirmed breast cancer who underwent surgical treatment at Guangzhou Red Cross Hospital between January 1, 2020, and December2023, 2023 were enrolled. Group differences in BCRL incidence were analyzed using the chi-square test. Univariate logistic regression was used to identify factors associated with BCRL.

**Results:**

BCRL occurred in 31 patients (15.4%). Univariate analysis revealed that tumor stage, axillary lymph node dissection, sentinel lymph node biopsy, chemotherapy, and regional nodal irradiation were significantly associated with BCRL (all P< 0.05). Taxane-based regimens (odds ratio [OR] = 4.018, 95% confidence interval [CI]: 1.162–13.890), long-course chemotherapy (OR = 4.887, 95% CI: 1.396–17.116), and the combination of neoadjuvant and adjuvant chemotherapy (OR = 4.50, 95% CI: 1.14–17.762) were significantly associated BCRL (all P< 0.05).

**Conclusion:**

Chemotherapy was associated with BCRL. Specifically, this association was observed with taxane-based regimens, long-course chemotherapy, and the combination of neoadjuvant and adjuvant chemotherapy. These findings highlight potential lymphotoxicity of specific regimens and may inform vigilant monitoring for BCRL in patients receiving such therapies, which could facilitate early intervention.

## Introduction

Breast cancer-related lymphedema (BCRL) is a common and debilitating complication following breast cancer treatment. A meta-analysis encompassing 72 studies and 29,612 women reported a pooled incidence of BCRL of 16.6%, with the majority of cases occurring within the first 24 months post-surgery (1). BCRL is characterized by the accumulation of protein-rich fluid in the interstitial spaces of the ipsilateral upper limb, leading to clinical manifestations such as swelling, discomfort, restricted mobility, and in severe cases, recurrent skin infections and progressive fibrosis, all of which seriously threaten patients’ quality of life (2). Established risk factors include extensive axillary surgery (axillary lymph node dissection (ALND) or sentinel lymph node biopsy (SLNB)), high body mass index (BMI), and regional nodal irradiation (RNI) (3).

Chemotherapy, a cornerstone of breast cancer management, is primarily administered in either adjuvant (postoperative) or neoadjuvant (preoperative) settings. Adjuvant chemotherapy is administered after tumor resection to eliminate micrometastases and reduce recurrence risk, whereas neoadjuvant chemotherapy is given before surgery, usually for patients with high-risk tumor biology or lymph node-positive diseases, aiming to reduce tumor volume and improve surgical outcomes (4). Identifying which aspects of chemotherapy correlate with BCRL risk represents a critical step toward developing targeted preventive strategies. The potential role of chemotherapy as a risk factor for BCRL remains controversial. Several studies have identified a significant association (5–7), while others report no significant correlation (8). A critical methodological gap in prior research lies in the oversimplified treatment of chemotherapy as a dichotomous exposure. This approach fails to dissect the distinct and potentially interactive effects of specific regimens, cumulative dosing, and temporal sequencing, thereby limiting the evidence base needed to guide clinicians in tailoring surveillance and preventive interventions for individual patients at varying risk levels.

This study aimed to investigate the association between chemotherapy and BCRL, with a specific focus on effects of different regimens, treatment duration (number of cycles), and timing of chemotherapy. These findings aim to provide novel insights for clinical risk stratification, timely intervention, and improved long-term management of breast cancer survivors.

## Materials and methods

### Ethical considerations

This study adhered to the STROBE (Strengthening the Reporting of Observational Studies inEpidemiology) guidelines (9). This single-center, retrospective cohort study was conducted at Guangzhou Red Cross Hospital. The study protocol was approved by the Institutional Review Board (Approval No: 2024-450-01), and the informed consent was waived the retrospective design and the use of anonymized clinical data. A copy of the ethical approval document is provided as **Supplementary Material**. All procedures were performed in accordance with ethical standards of the Declaration of Helsinki.

### Patient selection

A total of 296 patients with primary breast cancer who underwent surgery at Guangzhou Red Cross Hospital between January 1, 2020, and December2023, 2023 were initially identified. Inclusion criteria were (1): histopathological diagnosis of primary breast cancer (2); received comprehensive treatment. Exclusion criteria were (1): lack or loss of clinical or follow-up data (2); presence of other malignant tumors (3); upper limb edema caused by other reasons such as hypoproteinemia, compression from supraclavicular, cervical, or axillary lymphadenopathy, upper limb venous thrombosis, or tumor thrombus.

**Figure 1:**
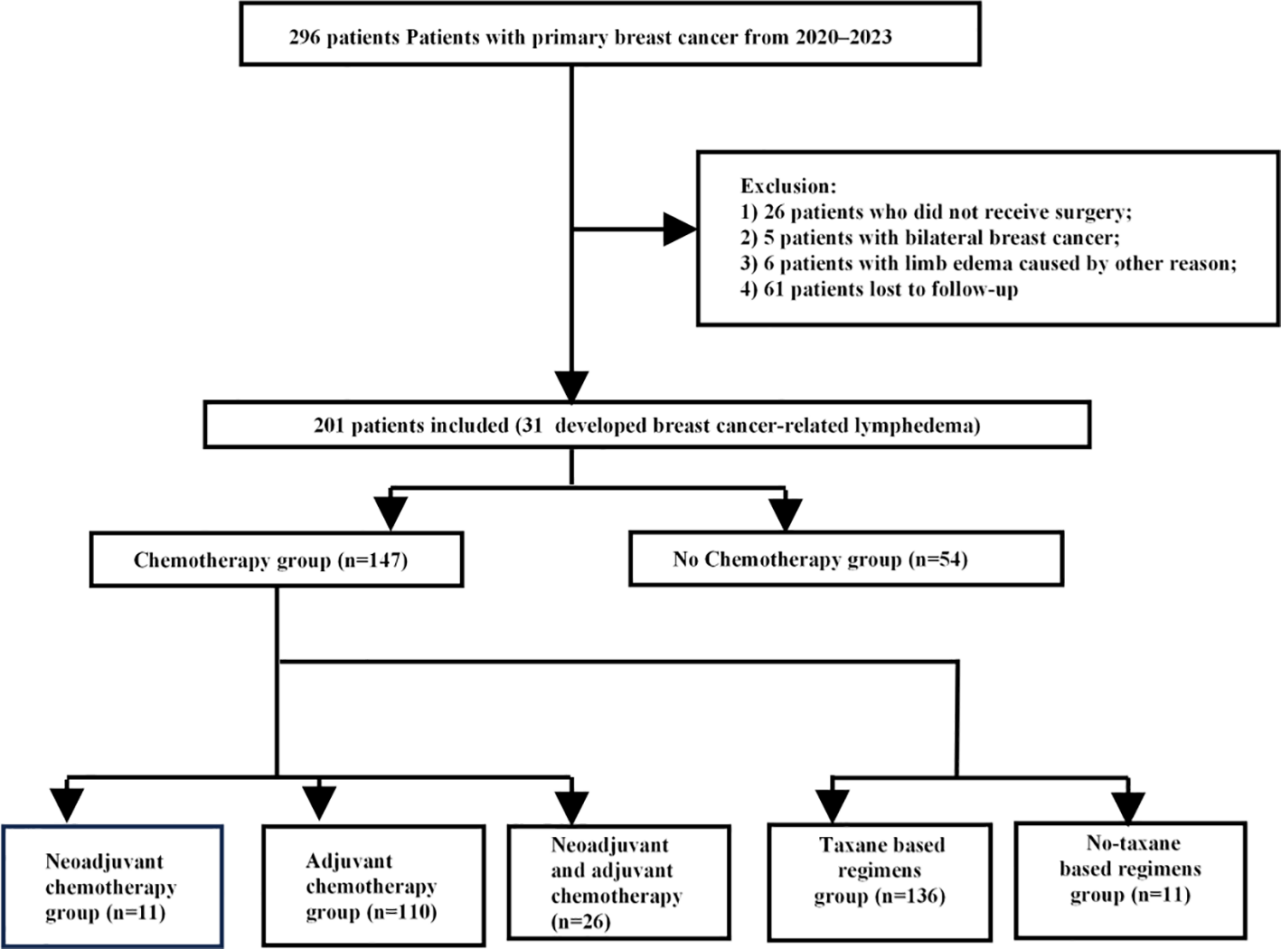
Flowchart of patient selection.

Patients were screened according to the following process, and excluded populations included: 26 patients who did not receive surgeries; 5 patients with bilateral breast cancer; 6 patients with limb edema caused by other reasons; 61 patients lost to follow-up. The remaining 201 patients were included in the present analysis. **Figure 1**. Flow diagram of the screen of patients.

### Data collection

The patient data was collected from the electronic health record, the following data was extracted: demographic characteristics (age and sex), medical history (other tumors), physical examination measurements (height and weight), pathology diagnosis, tumor stage, and treatment details. Treatment decisions followed the NCCN Clinical Practice Guidelines in Oncology for Breast Cancer. Extracted treatment variables included: type of primary surgery (breast-conserving surgery or mastectomy), lymph node surgical procedure (SLNB or ALND), chemotherapy, radiotherapy (chest wall/breast irradiation or RNI), hormonal therapy, targeted therapy, and duration of follow-up.

This study included 16 Stage IV breast cancer patients who underwent surgery, 7 of whom were initially staged as Mx (indicating those with uncertain distant metastasis at presentation). However, surgery was performed based on comprehensive multidisciplinary clinical judgments, with either limited metastatic burden or a good response to prior systemic therapy. This reflects the real-world clinical decision-making context.

Chemotherapy was administered with an implanted port orally or on the contralateral side. Treatment consisted of four to eight cycles. Patients were stratified into two groups based on the number of cycles received: the short-course group (1–4 cycles) and the long-course group (5–8 cycles). Regarding timing, patients were stratified into three groups: neoadjuvant chemotherapy, adjuvant chemotherapy, and the combination of neoadjuvant and adjuvant chemotherapy.

### Classification of chemotherapy regimens

Chemotherapy regimens were categorized based on the inclusion of taxane agents. The taxane-based group comprised all patients who received any regimens incorporating a taxane (docetaxel or paclitaxel), regardless of concomitant agents. Specifically, this included the following combination regimens: TC (docetaxel + cyclophosphamide), TX (docetaxel + capecitabine), GT (gemcitabine + paclitaxel/docetaxel), and TAC (docetaxel + doxorubicin + cyclophosphamide), as well as sequential anthracycline-taxane regimens (e.g., AC→T). The non-taxane group included patients who received cytotoxic regimens without any taxane, such as AC (doxorubicin + cyclophosphamide), EC (epirubicin + cyclophosphamide), or capecitabine monotherapy.

### BCRL diagnosis

Suspected BCRL was identified during routine follow-up visits either by clinician suspicion on physical examination or by patient self-report of limb swelling. For all suspected cases, a standardized limb circumference measurement was then performed to confirm the diagnosis.​ The measurement protocol was as follows: limb circumference measurements were taken by the attending clinical nurses, who were blinded to patients’ treatment details. BCRL was definitively diagnosed if the circumference exceeded that of the contralateral side by 2 cm or more at any measured point.

### Lymphedema staging

Lymphedema was staged according to the clinical staging system of the International Society of Lymphology (10).

Stage I (Mild): Early accumulation of fluid that is relatively high in protein content. Pitting edema is present. The swelling may be reduced with limb elevation.

Stage II (Moderate): Pitting edema progresses to non-pitting edema due to tissue fibrosis. Limb elevation alone rarely reduces swelling.

Stage III (Severe): The tissue is hard and fibrotic (sclerotic), and pitting is absent. Skin changes such as thickening, hyperkeratosis, and papillomas are present. This stage is also known as lymphostatic elephantiasis. Limb circumference was measured using a non-elastic tape measure.

### Statistical methods

All data were analyzed using SPSS software (version 26.0; IBM Corp, Armonk, NY, USA). Age, BMI and treatment characteristics were presented as mean ± standard deviation for continuous variables and as frequency (percentage) for categorical variables. Group comparisons were performed using an the unpaired, two-tailed t-test for continuous variables or the χ² test (or the Fisher’s exact test where appropriate) for categorical variables. Univariate logistic regression analyses were used to evaluate the association of treatment characteristics with BCRL. Subgroup analysis was performed to examine factors associated with BCRL severity (mild or moderate-to-severe vs. no) using multinomial logistic regression, Odds ratio (OR) with 95% confidence interval (CI) and P-values were calculated. A two-sided P-value < 0.05 was considered statistically significant for all analyses.

## Results

### General characteristics

During the follow-up period (median 60.1 months, range 12.0 – 140.2 months), of the 31 (15.42%) patients who developed BCRL, 17 (54.8%) were classified as having mild lymphedema (Stage I), 10 (32.3%) as moderate (Stage II), and 4 (12.9%) as severe (Stage III). The mean age was 60.10 ± 10.80 years, and the mean BMI was 21.94 ± 3.4. Regarding surgery, 26 (12.50%) patients underwent breast-conserving surgery, 175 (87.50%) patients underwent modified radical mastectomy. For lymph node surgery, SLNB and ALND were performed in 102 (50.7%) and 97 (48.3%) patients, respectively. Regarding radiotherapy, 60 patients (29.85%) received chest wall/breast irradiation, while 32 patients (15.92%) received RNI. Chemotherapy was administered to 147 patients (73.1%): 11 (5.5%) received no-taxane based regimens, and 136 (67.7%) received taxane-based regimens. Regarding chemotherapy timing, 11 patients (5.5%) received neoadjuvant only, 110 (54.7%) adjuvant only, and 26 (12.9%) the combination of neoadjuvant and adjuvant chemotherapy. Regarding treatment duration, 44 patients (21.9%) were in the short-course group and 103 (51.2%) in the long-course group. (**Table 1**).

**Table 1:** Comparison of baseline characteristics between groups.

Variable	Total (n=201)	Non-BCRL group (n=170)	BCRL group (n=31)	Statistical value	*P*
Age, Mean ± SD	60.10 ± 10.80	60.48 ± 10.78	57.52 ± 10.95	t=1.406	0.161
BMI (kg/m^2^)	21.94 ± 3.4	21.35 ± 5.2	20.56 ± 4.8	t=2.516	0.232
Surgery				χ²=2.134	0.144
Breast-conserving	26 (12.93%)	25 (14.71%)	1 (3.23%)		
Mastectomy	175 (87.07%)	145 (85.29%)	30 (96.77%)		
ALND				χ²=20.341	<.001
No	104 (51.74%)	100 (58.82%)	4 (12.90%)		
Yes	97 (48.26%)	70 (41.18%)	27 (87.10%)		
SLNB				χ²=22.829	<.001
No	99 (49.25%)	71 (41.76%)	28 (90.32%)		
Yes	102 (50.75%)	99 (58.24%)	3 (9.68%)		
Stage				χ²=16.922	0.001
I	59 (29.35%)	57 (33.53%)	2 (6.45%)		
II	85 (42.29%)	71 (41.76%)	14 (45.16%)		
III	41 (20.40%)	33 (19.41%)	8 (25.81%)		
IV	16 (7.96%)	9 (5.29%)	7 (22.58%)		
Chemotherapy				χ²=4.525	0.033
No	54 (26.86%)	51 (31.76%)	3 (9.68%)		
Yes	147 (73.13%)	119 (68.24%)	28 (90.32%)		
Radiotherapy				χ²=5.589	0.061
No	109 (54.23%)	98 (57.65%)	11 (35.48%)		
CWBI	60 (29.85%)	48 (28.23%)	12 (38.71%)		
RNI	32 (15.92%)	24(14.11%)	8 (25.81%)		
Hormonal therapy				χ²=0.01	0.978
No	45 (22.39%)	38 (22.35%)	7 (22.58%)		
Yes	156 (77.61%)	132 (77.65%)	24 (77.42%)		
Targeted therapy				χ²=1.08	0.743
No	160 (79.60%)	136 (80.00%)	24 (77.42%)		
Yes	41(20.40%)	34 (20.00%)	7 (22.58%)		

BCRL, Breast cancer-related lymphedema; ALND, Axillary lymph node dissection; SLNB, sentinel lymph node biopsy; CWBI, Chest wall/breast irradiation; RNI, regional nodal irradiation.

### Comparison between BCRL and non-BCRL groups

Factors such as tumor stage, ALND, SLNB, chemotherapy, and radiotherapy showed statistically significant differences between the BCRL and non-BCRL groups (P < 0.05). However, age, BMI, hormonal therapy, targeted therapy, and surgical approach did not show statistically significant differences between the groups (P > 0.05) (**Table 1**). Most patients had low body mass index (BMI; <22 kg/m^2^). Comparison of BCRL incidence rates using chi-square tests showed that incidence was significantly higher in the taxane-based group and long-course chemotherapy group compared to the no chemotherapy group (P < 0.05) (**Table 2**).

**Table 2:** Differences in BCRL incidence among different chemotherapy drugs and timing.

Groups	Total	Non-BCRL group	BCRL group	Comparison	χ²	*P*
Chemotherapy regimen						
no	54	51	3	Taxane vs. no	4.498	0.034
No-taxane based	11	9	2	No-taxane vs. no	0.659	0.417
Taxane based	136	110	26	No-taxanevs. taxane	0	1.000
Chemotherapy courses						
0	54	51	3	Short-course vs. no	0.454	0.051
Short-course (≤4)	44	39	5	Long-course vs. Short-course	1.746	0.186
Long-course (>4)	103	80	23	Long-course vs. no	6.051	0.014
Chemotherapy timing						
Neoadjuvant	11	8	3	Neoadjuvant vs. Adjuvant	0.168	0.682
Adjuvant	110	91	19	Adjuvant vs. Neoadjuvant+Adjuvant	0.165	0.685
Neoadjuvant+Adjuvant	26	20	6	Neoadjuvant vs. Neoadjuvant+Adjuvant	0.00	1.00

### Factors associated with BCRL

Univariable logistic regression analysis identified several factors significantly associated with BCRL (**Table 3**). Chemotherapy was also significantly associated with BCRL(OR = 4.0, 95% CI: 1.163 to 13.754, P = 0.028). Within chemotherapy-specific analyses, taxane-based regimens (OR = 4.018, 95% CI: 1.162 to 13.890, P = 0.028), long-course chemotherapy (OR = 4.887, 95% CI: 1.396 to 17.116, P = 0.013), and the combination of neoadjuvant and adjuvant chemotherapy (OR = 4.5, 95% CI: 1.14 to 17.762, P = 0.032) were all associated with BCRL. Among treatment factors, ALND and RNI demonstrated the strongest association with BCRL, with a relatively narrow CI indicating a precise estimate.

**Table 3:** Factors associated with BCRL in univariate logistic regression analysis.

Variable	β	S.E	Z	*P*	OR (95%CI)
Surgery	1.643	1.039	2.500	0.114	5.172 (0.675 ~ 39.663)
ALND	2.266	0.558	16.497	<.001	9.643 (3.230 ~ 28.784)
SLNB	-2.566	0.627	16.745	<.001	0.077 (0.022 ~ 0.263)
Chemotherapy (Yes vs. No)	1.386	0.630	4.840	0.028	4.0 (1.163 ~ 13.754)
Chemotherapy regimen (Ref: No Chemo)					
No-taxane based vs. No Chemo	1.329	0.982	1.832	0.176	3.778(0.551~ 25.882)
Taxane-based vs. No Chemo	1.391	0.633	4.830	0.028	4.018(1.162-13.890)
Chemotherapy courses (Ref: No Chemo)					
Short-course (≤4)	0.779	0.761	1.049	0.306	2.179(0.491-9.679)
Long-course (>4)	1.587	0.639	6.157	0.013	4.887(1.396-17.116)
Chemotherapy timing (Ref: No Chemo)					
Neoadjuvant vs. No Chemo	1.622	0.853	3.619	0.057	5.062(0.952~26.919)
Adjuvant vs. No Chemo	0.993	0.579	2.943	0.086	2.70(0.868~8.398)
Neoadjuvant+Adjuvant vs. No Chemo	1.504	0.701	4.610	0.032	4.5(1.14~17.762)
Stage (Ref: Stage I)					
Stage II vs. Stage I	1.726	0.777	4.942	0.026	5.620 (1.227 ~ 25.747)
Stage III vs. Stage I	1.933	0.820	5.552	0.018	6.909 (1.384 ~ 34.486)
Stage IV vs. Stage I	3.099	0.888	12.445	<.001	22.167 (3.963 ~ 123.982)
Hormonal therapy (Yes vs. No)	-0.013	0.467	0.001	0.978	0.987 (0.395 ~ 2.467)
Radiotherapy (Ref: No)					
CWBI	0.801	0.453	3.124	0.077	2.227(0.916 ~ 5.413)
RNI	1.088	0.517	4.424	0.035	2.970(1.077 ~ 8.188)
Targeted therapy (Yes vs. No)	0.154	0.470	0.107	0.743	1.167(0.464 ~ 2.933)

BCRL, Breast cancer-related lymphedema; ALND, Axillary lymph node dissection; SLNB, sentinel lymph node biopsy; CWBI, Chest wall/breast irradiation; RNI, regional nodal irradiation.

It is noteworthy that Stage IV disease exhibited an extremely high point estimate (OR = 22.167), but with an exceptionally wide CI (2.134 to 230.268), indicating very low precision. This is primarily attributable to the very small number of events in this subgroup (n=16), and the result should be considered as a highly uncertain, exploratory finding.

### Subgroup analysis

To further investigate the impact of different treatment factors on BCRL severity, and given the limited number of cases, the clinical stages were consolidated into two severity categories: mild (Stage I; n=17) and moderate-to-severe (Stages II-III combined; n=14). A multinomial logistic regression analysis was then performed, with BCRL severity (mild or moderate-to-severe) as the outcome and no BCRL serving as the reference group.

Subgroup analyses for BCRL severity are presented in **Table 4**. In the “Moderate-Severe vs. No Lymphedema” model, ALND and radiotherapy showed statistically significant associations (P< 0.05). However, all OR in these analyses were accompanied by exceptionally wide 95% CI, with several estimates approaching computational limits (e.g., OR = 4.893×10^−5^ for ALND; OR = 2.217×10^−9^ for CWBI). These extreme confidence intervals indicate substantial statistical uncertainty and suggest potential overfitting in models with limited event counts. Consequently, while these analyses identified nominally significant factors, the results must be interpreted with extreme caution and regarded as purely exploratory for hypothesis generation.

**Table 4:** Results of multinomial logistic regression for the subgroup analysis of lymphedema severity.

Variable	Mild vs. No Lymphedema		Moderate-Severe vs. No Lymphedema	
	OR (95%CI)	*P*	OR (95%CI)	*P*
Surgery	0.228(0.004 ~ 14.355)	0.484	0.312(0.006 ~ 16.007)	0.562
ALND	38230.435(7.381E-31 ~ 1.980E + 39	0.796	4.893E-5 (7.373E-6 ~ 0.000)	0.000
SLNB	144280.554(2.745E-30 ~ 7.583E + 39)	0.771	0.000	0.000
Chemotherapy (Yes vs. No)	1.087(0.012 ~ 100.024)	0.971	1.301(0.002~ 804.193)	0.936
Chemotherapy Regimen (Ref: No Chemo)				
No-taxane based vs. No Chemo	0.542(0.038~ 7.798)	0.653	1.058(0.105~ 10.692)	0.962
Taxane-based vs. No Chemo	0.544(0.094~ 3.140)	0.496	0.174(0.013~ 2.251)	0.181
Chemotherapy courses (Ref: No Chemo)				
Short-course (≤4)	2.627(0.279~ 24.761)	0.399	1.455(0.096~ 22.091)	0.787
Long-course (>4)				
Chemotherapy Timing (Ref: No Chemo)				
Neoadjuvant vs. No Chemo	0.689(0.009~ 51.720)	0.866	0.473(0.001~ 230.914)	0.813
Adjuvant vs. No Chemo	0.469(0.031~ 7.211)	0.587	1.354(0.060~ 30.711)	0.849
Neoadjuvant+Adjuvant vs. No Chemo	1.133(0.230~ 5.594))	0.878	3.102(0.413~ 23.281)	0.271
Stage (Ref: Stage I)				
Stage II vs. Stage I	0.480(0.028~ 8.201)	0.613	0.314(0.021~ 4.739)	0.403
Stage III vs. Stage I	0.652(0.085~ 5.001)	0.681	0.475(0.062~ 3.648)	0.474
Stage IV vs. Stage I	1.128(0.157~ 8.125)	0.905	0.091(0.007~ 1.174)	0.066
Hormonal therapy (Yes vs. No)	0.675(0.143~ 3.180)	0.620	0.900(0.175~ 4.644)	0.900
Radiotherapy (Ref: No)	0.381(0.087 ~ 1.666)	0.200	1.313(0.282~ 6.104)	0.728
CWBI	1.449E-8(2.695E-9 ~ 7.788E-8)	0.000	2.217E-9(4.018E-8 ~ 1.224E-10)	0.000
RNI	6.082E-9(1.664E-9 ~ 2.224E-8)	0.000	1.521E-9(1.512E-9 ~ 1.512E-9)	0.000
Targeted therapy (Yes vs. No)	1.446(0.296 ~ 7.068)	0.649	0.992(0.203 ~ 4.851)	0.992

BCRL, Breast cancer-related lymphedema; ALND, Axillary lymph node dissection; SLNB, sentinel lymph node; CWBI, Chest wall/breast irradiation; RNI, regional nodal irradiation.

## Discussion

The identification of risk factors is crucial for the early detection and prevention of BCRL. Our findings confirm that chemotherapy is associated with BCRL, particularly with taxane-based regimens. This aligns with prior literature indicating that chemotherapy, particularly taxanes, increases the risk of lymphedema (11). Zhu W et al. (12) reported a trend toward a significantly higher incidence of lymphedema with docetaxel compared to other agents. Similarly, Yuan et al. (5) identified taxane-based chemotherapy as an independent risk factor for BCRL. And in the study by Kim et al., the risk of lymphedema is 4.2-fold higher in patients who receive taxane-based chemotherapy than in patients who do not receive taxane-based chemotherapy (13). However, a large prospective cohort study by Swaroop et al. (14). concluded that neither docetaxel nor paclitaxel increased the risk of lymphedema, in this report, the cumulative incidence of lymphedema over 2 years was only 5.27%, which is notably lower than rates in other studies (5, 11, 12). Furthermore, evidence from Penn IW et al. (15) demonstrated that patients treated with taxane-based chemotherapy were more prone to develop persistent lymphedema, whereas those who did not receive chemotherapy or underwent non-taxane regimens tended to exhibit transient lymphedema. In addition, patients receiving paclitaxel were at a higher risk of developing persistent lymphedema than those who underwent treatment with docetaxel or other types of taxane.

Several studies have focused on elucidating how chemotherapy contributes to the pathogenesis of lymphedema. Mawaki A et al. (16) generated a docetaxel-induced edema formation model by the subcutaneous administration of docetaxel in the lateral tail vein of mice, and they focused on changes in vascular permeability, fluid retention in tissues, and interstitial fluid flow in lymph vessels caused by docetaxel administration. The principal mechanism is thought to be increased vascular endothelial cell permeability, resulting in the extravasation of plasma albumin into the surrounding tissues, the severity of edema is proportional to the cumulative dose of the drug administered. Another study found severe sclerosis of lymphatic vessels in limbs of patients developing lymphedema after docetaxel, possibly due to drug leakage into interstitial tissues directly damaging lymphatics (17). Additionally, chemotherapy-induced leukopenia and neutropenia, common complications, may increase the risk of arm infections and inflammation, potentially triggering or exacerbating lymphedema (18). These findings suggest a complex pathogenesis involving adipose tissue alterations and inflammatory factors, warranting further investigation.

The potential differential impact of chemotherapy timing on lymphedema risk continues to be a point of contention in the literature. Compared to no chemotherapy, our study found that the combination of neoadjuvant and adjuvant chemotherapy was associated with BCRL, neoadjuvant chemotherapy alone or adjuvant chemotherapy alone showed no significant association. This contrasts with some reports, Yan Huang et al. (19) also reported that the timing of chemotherapy significantly impacted BCRL risk, patients receiving neoadjuvant chemotherapy had a significantly higher risk (OR = 2.209) than those receiving adjuvant chemotherapy. Jung et al. (20) reported a higher BCRL risk in patients receiving neoadjuvant chemotherapy compared to adjuvant chemotherapy (HR = 1.39). Based on the data from a large cohort of 5549 breast cancer patients who underwent surgery, researches identified the use of adjuvant chemotherapy and neoadjuvant chemotherapy were significantly associated with lymphedema (11). Other studies involving 409 ALND patients found that neoadjuvant chemotherapy increased BCRL risk by 3.76 times and was an independent risk for BCRL (21). Another prospective cohort study of 266 ALND patients also identified neoadjuvant chemotherapy as a risk factor for BCRL (22). A retrospective analysis by Basta MN et al. of 3136 mastectomy patients reported a 10.4% BCRL prevalence, increased by neoadjuvant chemotherapy (23). Theoretically, neoadjuvant chemotherapy could reduce BCRL risk by decreasing the burden of positive lymph nodes (24). Conversely, a studies by Kim et al. (25), showed that neoadjuvant chemotherapy was not found to be a significant risk factor associated with BCRL, but the study did not include adjuvant chemotherapy patients. Similarly, Martínez-Jaimez1 P, et al. reported that neoadjuvant or adjuvant chemotherapy were not associated with lymphedema (8). Conversely, other studies found neoadjuvant and adjuvant chemotherapy were not significantly associated with an increased incidence of BCRL (26).

Our study identified that both long-course chemotherapy and the combination of neoadjuvant and adjuvant chemotherapy were significantly associated with BCRL. Potential reasons include: first, the extended duration of a long-course chemotherapy protocol inherently increases the cumulative exposure of the lymphatic vasculature and surrounding tissues to cytotoxic agents. Second, the strategy of administering chemotherapy both before and after surgery effectively doubles the chemotherapeutic exposure for a given patient, compared to a single chemotherapy. Consequently, these approaches result in a greater number of total treatment cycles and a higher cumulative dosage of potentially lymphotoxic drugs. This prolonged and intensified assault may overwhelm intrinsic repair mechanisms, leading to progressive and compounded damage to lymphatic endothelial cells, sustained inflammatory responses, and eventual fibrosis, which collectively impair lymphatic function and manifest as clinically significant BCRL. This is supported by a study of 950 patients with breast cancer, which identified chemotherapy as a lymphedema risk factor with a risk proportion of 38%. The chemotherapy regimen and the number of chemotherapy sessions increases the probability of lymphedema by 53% and 60%, respectively (27).

Our findings are directionally consistent with the established literature, suggesting that higher tumor stage may be associated with an increased risk of BCRL. In our analysis, stage IV disease showed a strong association with BCRL (OR = 22.167), albeit with an extremely wide confidence interval and a limited statistical stability. It is crucial to highlight that this estimate is derived from a very small number of Stage IV patients (n=16). More critically, Stage IV disease was almost invariably associated with more intensive local therapy, such as more extensive ALND and RNI. Our data confirmed both ALND and RNI as significant risk factors for BCRL in our cohort. As noted by Johnson et al., RNI following ALND can increase the risk of BCRL by 19.3% (27), and extensive radiotherapy can induce lymphatic dilation and fibrosis. Therefore, the observed association between Stage IV disease and BCRL is likely confounded, if not entirely mediated, by the greater intensity of local treatment that accompanies advanced disease, rather than representing an independent biological effect of the stage. Future studies with larger prospective cohorts that can control for such treatment differences are needed to clarify the relationship between disease stage and BCRL risk. Furthermore, the mean BMI in our cohort was relatively low. While this may reflect the demographic characteristics of our patient population, it may limit the direct generalizability of our findings to populations with a higher average BMI, where obesity itself is a known risk factor for lymphedema.

This study has certain limitations. First, BCRL was defined based on circumferential measurement using an objective threshold (a relative difference of ≥2 cm), this method may fail to identify early, mild, or subjective lymphedema where symptoms like heaviness precede or occur without reaching the circumferential diagnostic threshold. This could lead to an underestimation of the BCRL incidence, which would likely bias the observed effect estimates of risk factors. Second, the primary limitation of this study is the limited number of BCRL events, which precludes reliable multivariable analysis to control potential confounding factors. Consequently, the analyses were confined to univariable associations. While this approach minimizes the risk of overfitting, it also precludes an assessment of independent effects or complex interactions. The wide confidence intervals observed in the subgroup analyses (e.g., for ALND, SLNB, and radiotherapy) reflect substantial uncertainty, and the findings are at risk of overfitting. Thus, these results should not be interpreted as definitive conclusions. Their primary value lies in suggesting potential directions for future research rather than providing confirmatory evidence. Consequently, the results of the subgroup analyses require further validation in larger, prospective, independent cohorts. Finally, In this retrospective study, BCRL was assessed as a binary outcome based on circumferential measurement, circumference data required for precise volumetric analysis were lacking. Future prospective studies, if designed to systematically collect protocol-defined serial limb measurements, would be well-positioned to employ both volumetric assessment and time-to-event analyses (e.g., Kaplan-Meier method, Cox proportional hazards models). Such methodologies could help clarify the detailed temporal pattern of BCRL onset, identify periods of highest risk following specific treatments, and further refine clinical surveillance strategies.

## Conclusion

In this retrospective study, chemotherapy was associated with BCRL. Specifically, this association was observed with taxane-based regimens, long-course chemotherapy, and the combination of neoadjuvant and adjuvant chemotherapy. These findings highlight the need for clinicians to be aware of the potential lymphotoxic effects of specific chemotherapy protocols and to consider vigilant monitoring for early signs of BCRL during and after treatment, which could facilitate timely intervention and mitigate long-term morbidity.

## Data Availability

The raw data supporting the conclusions of this article will be made available by the authors, without undue reservation.

## References

[1] DiSipio T Rye S Newman B Hayes S . Incidence of unilateral arm lymphoedema after breast cancer: a systematic review and meta-analysis. Lancet Oncol. (2013) 14:500–15. doi: 10.1016/s1470-2045(13)70076-7. PMID: 23540561

[2] Armer JM Ballman KV McCall L Ostby PL Zagar E Kuerer HM . Factors associated with lymphedema in women with node-positive breast cancer treated with neoadjuvant chemotherapy and axillary dissection. JAMA Surg. (2019) 154:800–9. doi: 10.1001/jamasurg.2019.1742. PMID: 31314062 PMC6647005

[3] Matsumoto A Ushio K Kimura H Tomioka S Sasada S Asaeda M . Database study of risk factors for breast cancer-related lymphedema: a statistical analysis of 2359 cases over 10 years. Surg Today. (2025) 55:685–92. doi: 10.1007/s00595-024-02960-5. PMID: 39562356 PMC12011890

[4] Xie L Wang Y Wan A Huang L Wang Q Tang W . Research trends of neoadjuvant therapy for breast cancer: a bibliometric analysis. Hum Vaccin Immunother. (2025) 21:2460272. doi: 10.1080/21645515.2025.2460272. PMID: 39904891 PMC11801352

[5] Yuan Q Hou J Zhou R Liao Y Zheng L Jiao C . Development and validation of an intraoperative nomogram to predict breast cancer-related lymphedema based on the arm lymphatics distribution. Ann Surg Oncol. (2021) 28:7319–28. doi: 10.1245/s10434-021-09982-0. PMID: 33891201

[6] Altundag K . The association of taxanes with breast cancer-related lymphedema. J Surg Oncol. (2025) 131:105. doi: 10.1002/jso.27906. PMID: 39295566

[7] Jia M Pan L Yang H Gao J Guo F . Impact of neoadjuvant chemotherapy on breast cancer-related lymphedema after axillary lymph node dissection: a retrospective cohort study. Breast Cancer Res Treat. (2024) 204:223–35. doi: 10.1007/s10549-023-07183-9. PMID: 38097882

[8] Martinez-Jaimez P Armora Verdu M Forero CG Alvarez Salazar S Fuster Linares P Monforte-Royo C . Breast cancer-related lymphoedema: risk factors and prediction model. J Adv Nurs. (2022) 78:765–75. doi: 10.1111/jan.15005. PMID: 34363640

[9] White RG Hakim AJ Salganik MJ Spiller MW Johnston LG Kerr L . Strengthening the reporting of observational studies in epidemiology for respondent-driven sampling studies: “STROBE-RDS” statement. J Clin Epidemiol. (2015) 68:1463–71. doi: 10.1016/j.jclinepi.2015.04.002. PMID: 26112433 PMC4669303

[10] Executive Committee of the International Society of L . The diagnosis and treatment of peripheral lymphedema: 2023 consensus document of The International Society of Lymphology. Lymphology. (2023) 56:133–51. 39207406

[11] Byun HK Chang JS Im SH Kirova YM Arsene-Henry A Choi SH . Risk of lymphedema following contemporary treatment for breast cancer: an analysis of 7617 consecutive patients from a multidisciplinary perspective. Ann Surg. (2021) 274:170–8. doi: 10.1016/j.ijrobp.2019.06.2381. PMID: 31348041

[12] Zhu W Li D Li X Ren J Chen W Gu H . Association between adjuvant docetaxel-based chemotherapy and breast cancer-related lymphedema. Anticancer Drugs. (2017) 28:350–5. doi: 10.1097/cad.0000000000000468. PMID: 27997437

[13] Kim JS Kim JH Chang JH Kim DW Shin KH . Prediction of breast cancer-related lymphedema risk after postoperative radiotherapy via multivariable logistic regression analysis. Front Oncol. (2022) 12:1026043. doi: 10.3389/fonc.2022.1026043. PMID: 36387231 PMC9643832

[14] Swaroop MN Ferguson CM Horick NK Skolny MN Miller CL Jammallo LS . Impact of adjuvant taxane-based chemotherapy on development of breast cancer-related lymphedema: results from a large prospective cohort. Breast Cancer Res Treat. (2015) 151:393–403. doi: 10.1007/s10549-015-3408-1. PMID: 25940996 PMC4432026

[15] Penn IW Chang YC Chuang E Chen CM Chung CF Kuo CY . Risk factors and prediction model for persistent breast-cancer-related lymphedema: a 5-year cohort study. Support Care Cancer. (2019) 27:991–1000. doi: 10.1007/s00520-018-4388-6. PMID: 30105666 PMC6373263

[16] Mawaki A Kohta M Yoshimura A Nakatani T Nagao S Sugama J . Effect of docetaxel administration on fluid dynamics in mice. Fujita Med J. (2025) 11:59–63. doi: 10.20407/fmj.2024-023, PMID: 40309004 PMC12040483

[17] Fuse Y Karakawa R Yano T Yoshimatsu H . Lymph-venous anastomosis for breast cancer-related lymphoedema after docetaxel-based chemotherapy. J Clin Med. (2022) 11:1287. doi: 10.3390/jcm11051409. PMID: 35268500 PMC8910864

[18] Brown S Dayan JH Kataru RP Mehrara BJ . The vicious circle of stasis, inflammation, and fibrosis in lymphedema. Plast Reconstr Surg. (2023) 151:330e–41e. doi: 10.1097/prs.0000000000009866. PMID: 36696336 PMC9881755

[19] Najafali D Siotos C Kokosis G . Taxanes and breast cancer-related lymphedema. J Surg Oncol. (2025) 131:1000–1. doi: 10.1002/jso.28017. PMID: 39674951

[20] Jung SY Shin KH Kim M Chung SH Lee S Kang HS . Treatment factors affecting breast cancer-related lymphedema after systemic chemotherapy and radiotherapy in stage II/III breast cancer patients. Breast Cancer Res Treat. (2014) 148:91–8. doi: 10.1007/s10549-014-3137-x. PMID: 25253173

[21] Li X Huang H Lin Q Yu Q Zhou Y Long W . Validation of a breast cancer nomogram to predict lymphedema in a Chinese population. J Surg Res. (2017) 210:132–8. doi: 10.1016/j.jss.2016.11.009. PMID: 28457319

[22] Montagna G Zhang J Sevilimedu V Charyn J Abbate K Gomez EA . Risk factors and racial and ethnic disparities in patients with breast cancer-related lymphedema. JAMA Oncol. (2022) 8:1195–200. doi: 10.1001/jamaoncol.2022.1628. PMID: 35679026 PMC9185510

[23] Basta MN Wu LC Kanchwala SK Serletti JM Tchou JC Kovach SJ . Reliable prediction of postmastectomy lymphedema: the Risk Assessment Tool Evaluating Lymphedema. Am J Surg. (2017) 213:1125–33. doi: 10.1016/j.amjsurg.2016.08.016. PMID: 27745890

[24] Li F Lu Q Jin S Zhao Q Qin X Jin S . A scoring system for predicting the risk of breast cancer-related lymphedema. Int J Nurs Sci. (2020) 7:21–8. doi: 10.1016/j.ijnss.2019.12.007. PMID: 32099855 PMC7031125

[25] Kim M Park IH Lee KS Ro J Jung SY Lee S . Breast cancer-related lymphedema after neoadjuvant chemotherapy. Cancer Res Treat. (2015) 47:416–23. doi: 10.4143/crt.2014.079. PMID: 25544575 PMC4506114

[26] Clegg DJ Grande P Yoon J Onar G Watts J Khojandi A . The association of hand dominance with the development of breast cancer-related lymphedema after mastectomy: a retrospective analysis. Plast Reconstr Surg Glob Open. (2025) 13:e7251. doi: 10.1097/gox.0000000000007251. PMID: 41220877 PMC12599742

[27] Yaghoobi Notash A Yaghoobi Notash A Omidi Z Haghighat S . Prediction of lymphedema occurrence in patients with breast cancer using the optimized combination of ensemble learning algorithm and feature selection. BMC Med Inform Decis Mak. (2022) 22:195. doi: 10.1186/s12911-022-01937-z. PMID: 35879760 PMC9310496

